# Pegs not superior to screws for fixation of fractures of the proximal humerus

**DOI:** 10.1186/s13018-022-02947-3

**Published:** 2022-02-02

**Authors:** Ingrid Bønes, Anna Cecilie Karlberg, Maria Liljeholm, Alexander Nilsskog Fraser, Jan Erik Madsen, Tore Fjalestad

**Affiliations:** 1 Division of Orthopaedic Surgery (I.B., ML, J.E.M., T.F.), Oslo University Hospital, Postboks 4956, 0424 Nydalen, Oslo, Norway; 2 Division of Radiology and Nuclear Medicine, Department of Musculoskeletal Radiology (A.C.K.), Oslo University Hospital, Oslo, Norway; 3 Orthopaedic Department, Diakonhjemmet Hospital, Oslo, Norway; 4 Institute of Clinical Medicine, Faculty of Medicine, University of Oslo, Oslo, Norway

## Abstract

**Background:**

Angular stable plates were introduced two decades ago as a promising treatment for fixation of displaced fractures of the proximal humerus (PHF). However, high rates of adverse events and reoperations have been reported. One frequent reason is secondary penetration of screws into the glenohumeral joint, due to sinking of the fracture or avascular head necrosis. To prevent joint penetrations angular stable plates with smooth locking pegs instead of locking screws have been developed. The aim of the present study was to investigate whether blunt pegs instead of pointed screws reduced the risk of secondary penetration into the glenohumeral joint during fracture healing after operatively treated PHFs.

**Methods:**

From two different patient cohorts with displaced PHFs (60 treated with PHILOS plate with screws and 50 with ALPS-PHP plate with pegs), two groups were matched according to fracture type AO/OTA 11-B2 and 11-C2 and age (55–85 years). They were followed up at 3, 6 and 12 months. Primary outcome was radiographic signs of peg or screw penetrations into the glenohumeral joint at 12 months. Secondary outcomes were Oxford shoulder score (OSS) and Constant Score (CS) and radiographic signs of avascular humeral head necrosis (AVN).

**Results:**

Eighteen PHILOS patients with B2 and C2 fractures could be matched with a corresponding group of 18 operated with ALPS-PHP with pegs. The number of penetrations of pegs and screws were equal between the two groups and the development of avascular head necrosis did not differ either. The functional outcomes for both OSS and CS at 12 months was clearly in favor of patients without joint penetrations in both groups.

**Conclusion:**

We found no differences in the number of screw or peg penetrations in the PHILOS and ALPS-PHP group and the occurrence of AVN was equal. Joint penetrations led to inferior functional outcomes at 1 year.

The ClinicalTrials.gov identifier 20/11/12 prospectively for the Philos Group is NCT01737060, and for the ALPS group 11/03/20 retrospectively is NCT04622852.

## Background

Operative treatment for proximal humeral fractures (PHF) has been a challenge for decades [1–3]. The majority of these fractures are regarded as stable and treated non-operatively, while about 20% are severely displaced and managed operatively [4].

**Fig. 1:**
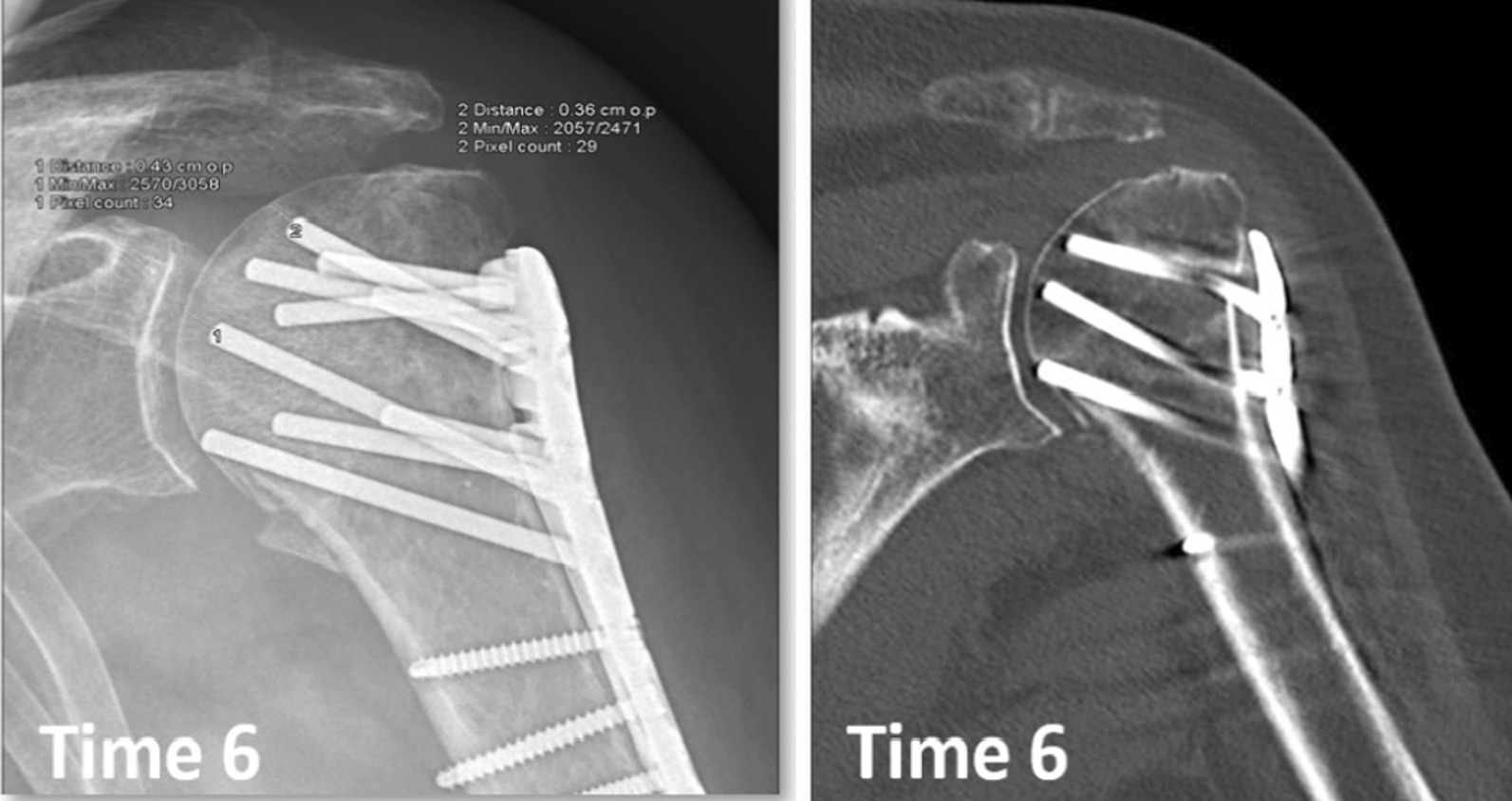
Proximal humeral fracture operated with an ALPS-PHP. At 6 months, radiograph gives suspicion of peg perforation, and CT scan confirms this

Open reduction and internal fixation (ORIF) with an angular stable plate is a frequently used option for the displaced fractures [5–7], despite reports of significant incidences of adverse events, especially with the 3 and 4 part fractures (AO/OTA types B and C) [8, 9]. Up to one third of operated patients is reported in need of secondary surgery [10]. Secondary penetration of locking screws into the glenohumeral joint is a frequent reason for this. Sinking (Fig. 1) may cause screw penetration of the humeral head during fracture healing, with or without radiographic signs of avascular head necrosis (AVN) [11, 12]. Aiming to reduce this risk of secondary implant penetration into the glenohumeral joint, smooth pegs have been introduced to replace screws, claiming that the tip of a traditional screw may penetrate the subchondral bone more easily than a cylindrical device with a blunt tip. Also, pegs may potentially reduce the severity of damage to the glenoid surface if penetrations occur [13].

The aim of this study was to investigate whether the use of blunt pegs instead of pointed screws reduced the risk of penetration into the glenohumeral joint during fracture healing after operatively treated PHFs.

## Methods

We compared radiographic results, functional outcome and adverse events from two patient cohorts with severely displaced proximal humeral fractures operated with either an implant utilizing ordinary angular stable screws or an implant with blunt pegs.

The angular stable implant “Anatomical Locking Plate System-Proximal Humeral Plating” (ALPS-PHP) [14] (Zimmer-Biomet, Warsaw, Indiana, USA) designed for blunt locking pegs in the humeral head and the “Proximal Humeral Internal Locking System” PHILOS plate (Synthes Solothurn, Switzerland) made for traditional locking screws were used for osteofixation in two patient cohorts operated during 2013–2019.

### Inclusion criterion

Patient operated with open reduction and internal fixation using a plate for a fracture type classified according to AO/OTA groups 11-B2 and 11-C2 equal to three- and four-parts fractures [15]. Displacement with respect to inclination had to be ≥ 45° in valgus or ≥ 30° in varus in a true AP radiograph, or ≥ 45° in the scapula Y projection with neutral arm rotation and/or contact between head fragment and shaft ≤ 50% in any projection. The degree of displacement of major and minor tubercle was not critical.

### Exclusion criteria

Radiographic exclusion criteria were patients with a radiographic sign of primary penetration of a screw or peg, i.e., perioperative penetration of the humeral head due to extensive drilling with the use of too long screw or peg, visible at the first postoperative radiographic examination. Furthermore, patients with a head split and fracture dislocations.

General exclusion criteria were age younger than 55 or older than 85. Patient should be asymptomatic in both shoulders prior to the injury, and suffer a mono-trauma. No alcohol or drug abuse, dementia, neurological diseases, or severe cardiovascular or lung diseases that would contraindicate surgery. Furthermore, patient should be compliant to rehabilitation.

### Patients

Patients were recruited from January 2013 to June 2019. In the period from January 2013–June 2017, 60 patients with B2- and C2-type fractures were treated with ORIF with the PHILOS plate. From August 2017–June 2019, 50 consecutive fractures with displaced A-, B- and C-type fractures were treated with the ALPS-PHP. Our study patients were selected from these two baseline cohorts (12/18 of PHILOS and 18/18 ALPS-PHP from one hospital, six PHILOS from three collaborating hospitals). All surgeons were experienced in the operative treatment, and the approach was deltopectoral for all patients.

As fracture type and age predict functional outcome and risk of complications, two groups (PHILOS and ALPS-PHP) were matched according to fracture types B2 or C2, and age groups 55–74 or 75–85 years. Eighteen patients were eligible for precise matching in both groups at 12 months.

For all patients in both groups, the injury type was a low energy trauma. ALPS-PHP group: Indoor 3/ Outdoor 13. PHILOS group: Indoor 4/ Outdoor 14. Details about the patients are shown in Table 1.

**Table 1:** Patient demographics

Patient group	Age group	Mean/median age	Patients (female)	Smoking	Premobidity^a^	ASA group^b^
ALPS-PHP	55–70	60.4/61	11(6)	1	5	2.0
ALPS-PHP	71–85	77.5/76.5	7 (5)	0	5	2.0
PHILOS	65–70	67.5/68	11(7)	1	4	1.9
PHILOS	71–85	76.8/77	7(5)	1	5	2,6

^a^Pre-mobidity means: Medical treatment for 1) Heart disease, 2) Blood pressure, 3) Diabetic disease or 4) Obstuctive lung disease

^b^Mean value

The Ethics Committee in the South East Health authority (REK 2017/681) approved the trial. The study registration number in http://www.ClinicalTrials.gov is NCT04622852.

### Surgical technique

For all operations, a senior consultant with skeletal trauma experience took part. For the PHILOS group, one surgeon (TF) took part in nine operations, and three took part in nine operations. For the ALPS-PHP group, one surgeon (TF) took part in 14 operations, and three surgeons in four operations.

The surgical technique was identical in both groups. A deltopectoral approach in the beach-chair position was used, with non-resorbable sutures to secure the rotator cuff and tubercle fragments. Both plates were placed along the axis of the humeral shaft and slightly posterior to the bicipital groove to prevent injury to ascending branch of the anterior humeral circumflex artery.

For both plates, all available pegs and screws were used, eight locking screws for the PHILOS plate and nine locking pegs for the high-type ALPS-PHP plate. Peg and screw lengths were chosen to leave 6–10 mm of bone toward the subchondral line, to compensate for sinking of the humeral head at the fracture site during healing [16]. Screw or peg support toward the calcar was given priority [17, 18].

The postoperative guidelines for instructed physiotherapy and self-exercises did not differ between the two groups. From the first postoperative day, patients were instructed to start standardized exercise program supported by a physiotherapist: Pendulum exercises, passive and active assisted exercises until four weeks. Functional exercises and isometric resistance with shoulder in neutral position from four to six weeks. Active dynamic strengthening exercises were introduced after six weeks, weight-bearing at eight weeks, while stretching at ten weeks.

### Outcomes

All radiographs were rated by an orthopedic surgeon (IB) and a radiologist specialized in skeletal injuries (ACK). The plain radiographic projection were standardized as the true front that provides a precise glenohumeral clear-space and the scapula Y-projection in a 90 degree angle to the true front projection. We aimed that tip of coracoid process should be at the same position compared to the humeral head for all examinations. These projections were identical for both the primary examination and the follow-ups within both groups.

Primary outcome was the difference between the radiographically verified number of penetrating pegs or screws through the subchondral bone of the humeral head at 1 year (secondary penetration due to sinking or AVN of the humeral head). Radiographs were evaluated after surgery (time 0) and at 3, 6 and 12 months. Cutout of screws or pegs was defined as a broken subchondral line.

**Fig. 2:**
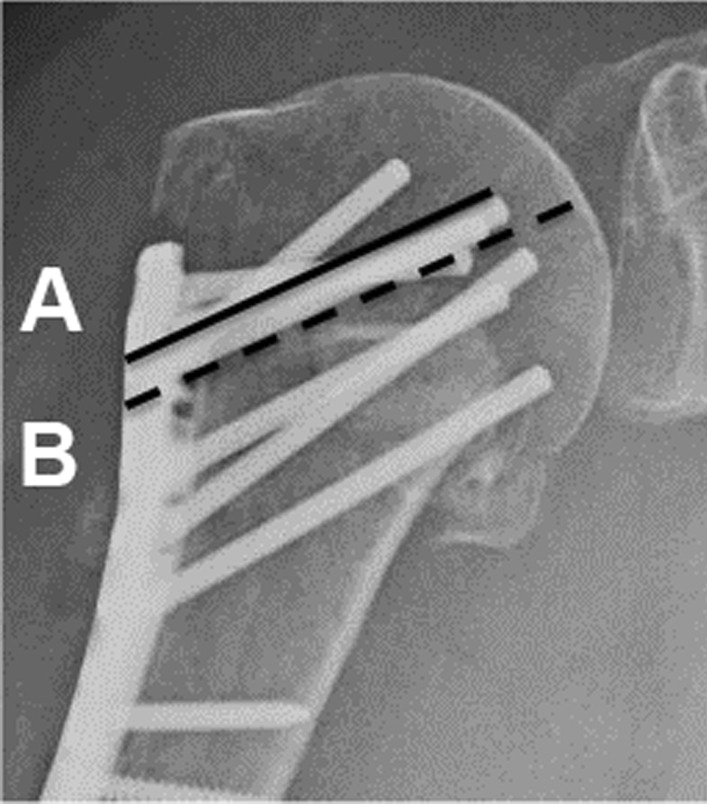
Measurement of ratio A/B: Peg or screw length = A and Plate subchondral distance = B

To explore whether the lengths of pegs and screws for either ALPS-PHP or PHILOS could be compared, three screws or pegs with a short distance to the subchondral surface were measured at time 0 on coronal radiographs in each patient (Fig. 2, length A). Length A was compared to the distance between the plate and the subchondral line (Fig. 2, distance B). Based on these three measurements, the ratios A_1,2,3_/B_1,2,3_ were calculated. For ALPS-PHP group, the calculated ratio was 0.82–0.86, while for PHILOS group 0.84–0.85 (mean values).

In the ALPS-PHP group, subchondral peg penetration were confirmed with a CT scan, however in the PHILOS group only occasionally.

Secondary outcomes were radiographic signs of avascular necrosis of the humeral head and clinical outcome measures. Constant score (CS) (0–100 points, 100 best) was measured by an independent physiotherapist at 12 months (CS12) and the self-assessment form Oxford Shoulder Score (OSS) (0–48 points, 0 best) at 3, 6 and 12 months [19, 20]. Additional adverse events registered were implant failure, infection, non-union and re-operations due to any cause.

**Fig. 3:**
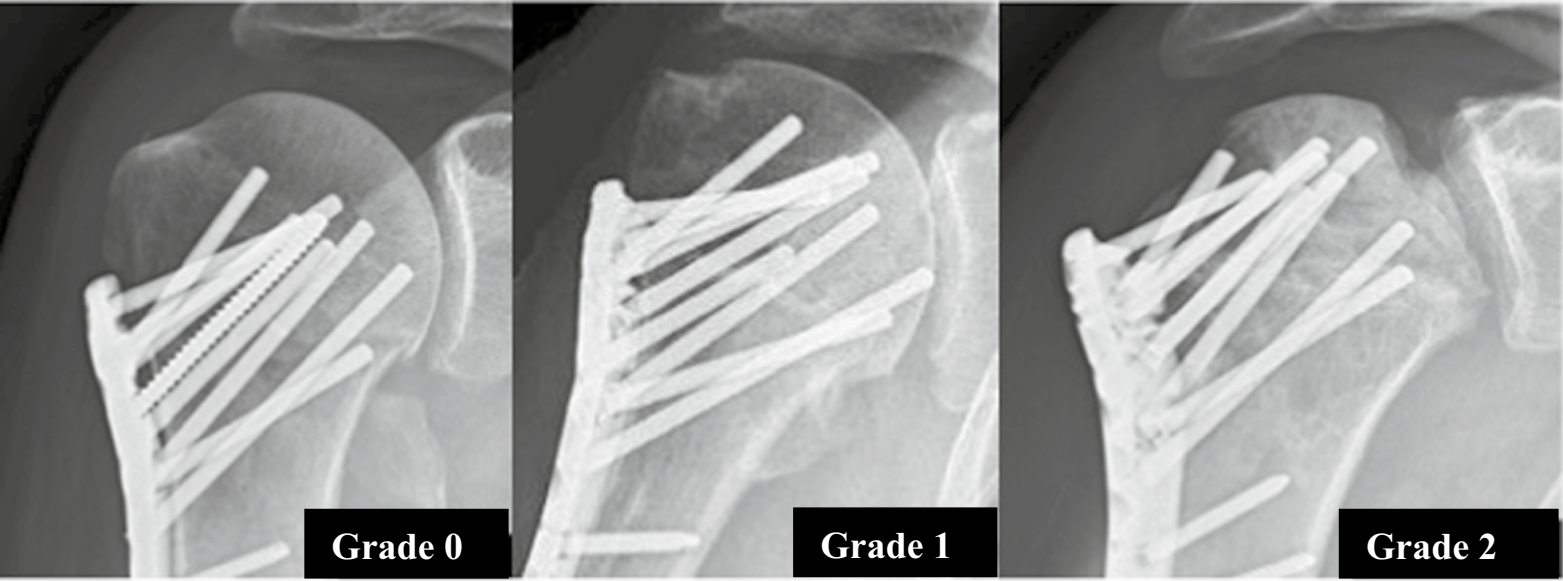
Method for classification of humeral head avascular necrosis at 12 months. Normal (= 0), less than 50% head area with trabecular change (= 1), more than 50% and/or segmental collapse of humeral joint surface (= 2)

AVN was classified according to a simple definition based on the AP radiograph (Fig. 3) [21–23]

Grade 0: Normal trabecular bone structure of the humeral head
Grade 1: Less than 50% change of trabecular bone structure observed in the head without evidence of segmental collapse
Grade 2: More than 50% change of trabecular bone structure in the head and/or segmental collapse of joint surface

### Follow-up

All patients were followed up at 3, 6 and 12 months with physical examination, interview and radiographs. At the final 12-month follow-up, an independent physiotherapist performed the Constant scorings.

## Statistics

Statistical analysis sample size was calculated from the primary outcome: Penetration of screw or peg into the glenohumeral joint. Provided a normal distribution of the distances between the tip of a screw or peg to the subchondral bone, the estimated mean difference between the groups can be set to 1 × standard deviation (SD). The level of significance (alpha) was 0.05. To achieve a power of 0.80, the number of patients required for each group was 17.

Mean values, median values and standard deviations were calculated. 95% confidence intervals (95% CI) were estimated with bootstrapping due to the small number of patients.

We calculated Odds Ratio (OR) for the probability of penetration (primary outcome) or developing AVN, by using logistic regression analysis.

Functional outcome OSS and CS were compared with independent t test and Mann–Whitney U test as appropriate. Statistical analyses were performed with SPSS version 25 (IBM).

## Results

### Radiographic results

In both groups, there were eleven B2-type and seven C2-type PHF. Group ALPS-PHP consisted of 18 patients aged 55–85 with B2- and C2-type fractures. In this group, twenty-two pegs penetrated nine humeral heads at 12 months, of which six had developed AVN of grade 1 (one patient) or grade 2 (five patients). In the PHILOS group, there were 18 screw penetrations in seven humeral heads, of which five had AVN, all grade 2. Taken into account the possible number of peg penetrations in group ALPS-PHP (18 plates times 9 pegs), the incidence of penetrations was 13.5%, while for group PHILOS (18 plates times 8 screws), 12.5% of the screws had penetrated into the joint. All patients with signs of AVN had penetrating screws or pegs. Two PHILOS and three ALPS-PHP plates had penetrating screws without signs of AVN (Table 2).

**Table 2:** Comparison of penetration of pegs or screws within age group, fracture group and numbers of avascular head necrosis (AVN) grade 1 or 2

Implant	Age group	Fracture group (B2/C2)	Penetrations (B2/C2)	Number SP/PP (B2/C2)	AVN (B2/C2)
ALPS-PHP	55–70	11 (6/5)	5 (2/3)	13 (3/10)	4 (2/2)
ALPS-PHP	71–85	7 (5/2)	4 (3/1)	9 (8/1)	2 (2/0)
PHILOS	65–70	11 (6/5)	4 (1/3)	6 (1/5)	3 (1/2)
PHILOS	71–85	7 (5/2)	3 (2/1)	12 (11/1)	2 (1/1)

SP, screw penetration; PP, peg penetration

The risk for penetration within the ALPS-PHP group was OR 1.57 compared to the PHILOS group (95% CI 0.418, 5,903). The respective OR for AVN was OR 1.3 (95% CI 0.313; 5.393, p-value 0.718). No statistical difference for OR between the ALPS-PHP and PHILOS group was found, although the first had somewhat higher risk of penetration.

### Clinical results

The functional outcomes differed significantly between patients with and without peg/screw penetrations, for both OSS12 (*p* = 0.002) and CS12 (*p* = 0.003). Mean OSS12 in ALPS without penetration was 9.8, with penetration 16.6. Mean CS12 in ALPS without penetration was 66.9, with penetration 44.6. Mean OSS12 in PHILOS without penetration was 7.8, with penetration 18.4. Mean CS12 in PHILOS without penetration was 71.4, with penetration 41.4. 95% bootstrapped CIs were (− 14.3, − 1.3) and (11.2, 39.9), respectively (Table 3).

**Table 3:** Constant score (CS) and Oxford Shoulder Score (OSS) at 12 months for 18 patients with ALPS-PHP and 18 patients with PHILOS classified according to penetration of humeral head or not. Both mean and median values given. Right column displays p-values of the difference between implants

Implant	Patients	Perforation Yes/No	CS 12 months Mean (Median)	OSS 12 months Mean (Median)	Difference (p) CS/OSS
ALPS-PHP	9	No	66.9 (73.3)	9.8 (8.0)	0.849/0.616
PHILOS	11	No	71.4 (72.2)	7.8 (6.0)	
ALPS-PHP	9	Yes	44.6 (44.4)	16.0 (15.0)	0.832/0.740
PHILOS	7	Yes	41.4 (37.3)	18.4 (16.0)	
ALPS + PHILOS	20	No	69.3 (72.8)	8.7 (6)	0.003/0.002
ALPS + PHILOS	16	Yes	43.0 (42.3)	16,7 (15.5)	

**Fig. 4:**
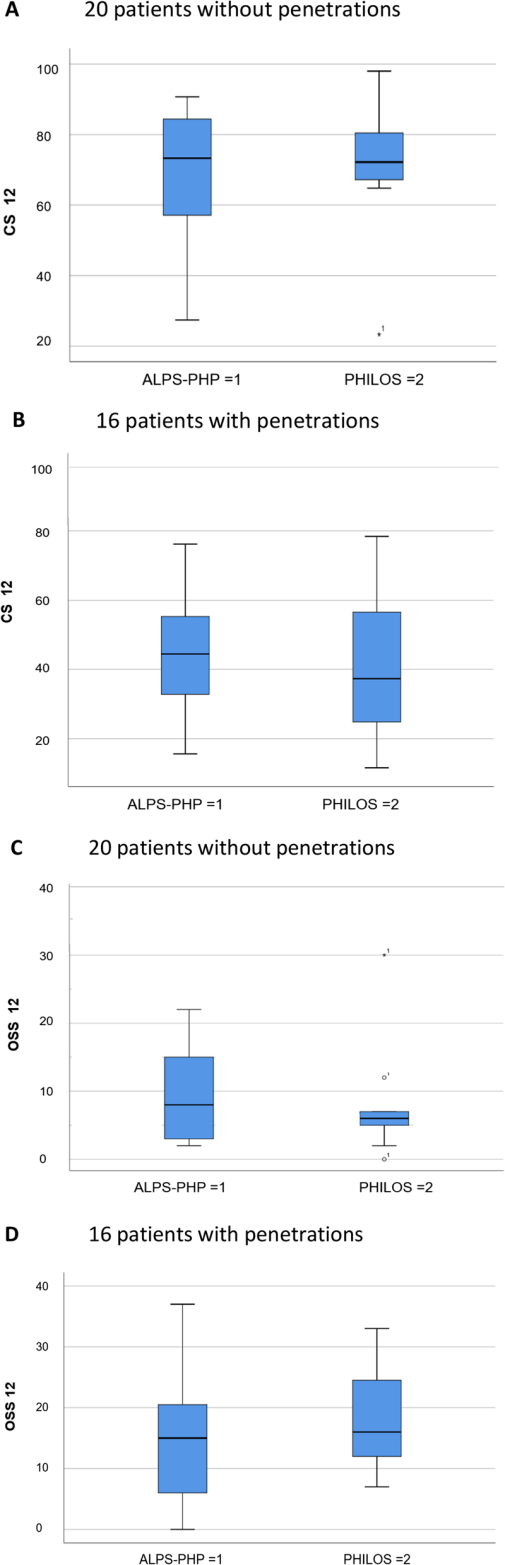
Constant Score and Oxford Shoulder Score at 12 months (CS12 and OSS12) with or without humeral head penetration of pegs or screws. ALPS-PHP = peg group, PHILOS = screw group. **A**, **B** CS at 12 months in patients without (20) and with (16) joint penetrations. **C**, **D** OSS at 12 months in patients without (20) and with (16) joint penetrations

For ALPS-PHP, the difference in OSS12 between the patients with penetration and no penetration was median 7, while for PHILOS 10, thus in favor of pegs. For CS12, the respective median difference was 28.9 and 34.9, thus 6.0 in favor of pegs (Fig. 4).

There were few other adverse events. One patient had the implant removed due to localized pain, despite no penetration of pegs. Another experienced loosening of a locking peg, which was consequently removed. There were no non-unions and no infections.

## Discussion

In this study, the use of pegs did not significantly reduce the frequency of penetrations into the glenohumeral joint compared with screws. The patients suffering peg or screw penetrations had a worse clinical outcome measured with OSS and CS at 1 year.

No patients with a primary screw- or peg penetration were included. Our aim was to examine a secondary penetration during follow-up at 1 year. The secondary penetration is due to either “sinking” of the humeral head during healing of the fracture (bone necrosis or remodeling) or to severe AVN with segmental collapse of the head [24, 25].

To the author’s knowledge, only two clinical reports comparing the use of pegs and screws in PHF have been published [26, 27]. One preliminary report concluded that the results were equal to other available plating systems for 15 patients after 7 months, while the most recent report concluded with fewer adverse events with pegs compared to threaded screws, however no differences between screw- and peg penetrations. The included groups were heterogeneous regarding fracture types and younger age, and included simple 2-part fractures. In our present study, we have compared severely displaced B2- and C2-type fractures in patients aged 55 to 85 in separate groups, which may give more precise information about a patient group with a high risk for failures and complications.

Several biomechanical studies have compared the use of pegs and screws in PHF, with inconsistent results. Schumer [28] compared pegs and screws in a 2-part fracture model in cadavers and could not detect strength differences between the two implant types. Yamamoto [29] performed a fairly similar study, and concluded that pegs showed slightly superior biomechanical characteristics compared to screws, even though no cutouts were observed in neither group. Recently, threaded pegs was also claimed to effectively increase the varus bending stiffness in the plate/peg construction in a finite element study [30]. However, there is an obvious lack of available clinical data on the use of pegs in PHFs.

In distal radius fractures, volar angular stable plates with locking pegs have been extensively used for the last decade, mostly motivated by the fact that the pegs have a smoother surface toward the extensor tendons in cases of accidental posterior cortical perforations. Clinically, however, the peg constructs do not seem to improve the results of distal radius fracture treatment [31].

The present study design was retrospective and contain inherent weaknesses. For one, we used the AO/OTA fracture classification to compare groups, an experienced skeletal radiologist and an orthopedic surgeon performed the interpretations of radiographs. Low intra-observer reproducibility has been reported, although somewhat better for those experienced with the method [32]. Thus, some inconsistencies concerning fracture classification should be taken into account.

Another weakness of the study was that the PHILOS patients did not have a routine CT scan if sign of screw penetrations were observed. This could potentially affect the precise number of screw penetrations. However, only two of the CT scans in the ALPS-PHP group resulted in a higher number of penetrations diagnosed compared to the plain radiographs. Another confounder for precise measurements of pegs or screws is the quality of radiographs, depending on a precise angulation of the X-ray beam and an antegrade plate projection, as shown in the Fig. 3 radiographs. This was taken into account with standardized radiographic examinations for all included patients [33].

Also, AVN may be difficult to detect and classify, and 12-month follow up is clearly too short to capture the complete numbers of this complication. In our series, AVN changes were observed in eight out of nine patients within six months. Some have reported development of AVN up to 5 years after injury, but these cases are rare [34, 35]. It is also claimed that the use of a deltopectoral approach might increase the risk of AVN, compared to the minimally invasive anterolateral approach [36]. In addition, surgery later than 48 h after has shown to increase the risk for developing AVN [37]. Only three out of 36 of our fractures were operated within 48 h.

Several different classification systems for AVN of the humeral head exist [38]. In this study, we used a simple definition with only three modalities, based on the true AP radiograph (Fig. 3) [21, 23]. Thus, our number of AVN of the humeral head may differ from other reports due to classification bias, but we are experienced with this classification system from our former studies.

The radiographic findings in this study do not support that pegs are better than screws, neither for radiographic results, nor the clinical outcome measured with OSS and CS at 1 year. We observed a slight, but non-significant difference between pegs and screws for the patients with radiographic signs of joint penetration. The idea that penetration into the glenohumeral joint was better tolerated with pegs than with screws could not be confirmed. Due to the relatively small number of patients, these findings should nevertheless be interpreted with caution, but call for further investigations.

## Conclusion

Fracture fixation with angular stable locking plate and pegs did not reduce the frequency of penetrations to the glenohumeral joint when compared to screws. We found a non-significant OR for penetration into the glenohumeral joint of 1,57 for ALPS-PHP compared to PHILOS group. The occurrence of AVN was almost equal, with a difference in OR 1,3. The patients who had screw or peg penetrations to the glenohumeral joint had significantly lower OSS and CS at 1 year.

## Data Availability

The datasets used analyzed during the current study are available from the corresponding author on reasonable request.

## Abbreviations

ALPS-PHP: Anatomical Locking Plate System-Proximal Humeral Plating
AO/OTA: Arbeitsgemeinschaft für Osteosynthesefrage /Orthopaedic Trauma Association
AVN: Avascular humeral head necrosis
CS: Constant Score
OSS: Oxford shoulder score
PHF: Proximal Humeral Fracture
PHILOS: Proximal Humeral Internal Locking System
